# Reversible Tuning Electrical Properties in Ferroelectric SnS with NH_3_ Adsorption and Desorption

**DOI:** 10.3390/nano14201638

**Published:** 2024-10-12

**Authors:** Wanqian Wang, Wei Luo, Sen Zhang, Chayuan Zeng, Fei Xie, Chuyun Deng, Guang Wang, Gang Peng

**Affiliations:** College of Science, National University of Defense Technology, Changsha 410073, China; wangwanqian22@nudt.edu.cn (W.W.); zhangsen@nudt.edu.cn (S.Z.); zengchayuan23@nudt.edu.cn (C.Z.); xiefei0314@nudt.edu.cn (F.X.); dengchuyun@nudt.edu.cn (C.D.); wangguang@nudt.edu.cn (G.W.)

**Keywords:** ferroelectric SnS, NH_3_ adsorption, NH_3_ desorption, air molecules, IV hysteresis

## Abstract

Two-dimensional (2D) ferroelectrics usually exhibit instability or a tendency toward degradation when exposed to the ambient atmosphere, and the mechanism behind this phenomenon remains unclear. To unravel this affection mechanism, we have undertaken an investigation utilizing NH_3_ and two-dimensional ferroelectric SnS. Herein, the adsorption and desorption of NH_3_ molecules can reversibly modulate the electrical properties of SnS, encompassing I–V curves and transfer curves. The response time for NH_3_ adsorption is approximately 1.12 s, which is much quicker than that observed in other two-dimensional materials. KPFM characterizations indicate that air molecules’ adsorption alters the surface potentials of SiO_2_, SnS, metal electrodes, and contacts with minimal impact on the electrode contact surface potential. Upon the adsorption of NH_3_ molecules or air molecules, the hole concentration within the device decreases. These findings elucidate the adsorption mechanism of NH_3_ molecules on SnS, potentially fostering the advancement of rapid gas sensing applications utilizing two-dimensional ferroelectrics.

## 1. Introduction

With the development, two-dimensional ferroelectric materials show great application potential in the manufacturing of micro ferroelectric components [1,2,3,4,5,6,7,8], and a large number of room-temperature two-dimensional ferroelectric materials have been discovered, such as 1L d1T-MoTe_2_ [9], 2L/3L WTe_2_ [10,11], α-In_2_Se_3_ [12,13,14,15], MX (M=Sn, Ge, X=S, Se, Te) [16,17,18,19], CuM^3+^P_2_Q_6_ (Q=S, Se) [20,21,22], etc. Among them, SnS has attracted much attention due to its stable in-plane anisotropic ferroelectric properties [17], and even monolayer SnS thin films still have in-plane ferroelectric properties at room temperature [23]. However, in the atmosphere, perfect ferroelectric domain images of SnS or other two-dimensional ferroelectric materials are always difficult to obtain with piezoresponse force microscopy (PFM) characterization [24,25,26,27]. Moreover, many two-dimensional materials are sensitive to lots of gas molecules, such as NH_3_, H_2_S, NO, etc. [28,29,30,31,32,33]. Most of the works focus on improving gas sensing performance parameters, such as increasing device response singularity, responsivity, and response speed [33]. In order to improve the responsivity of the device, most gas sensors prefer to use polycrystalline or colloidal materials to increase the contact area [30,31]. Few studies have used single crystal thin-film materials to prepare gas sensors, resulting in a lack of in-depth analysis and understanding of the mechanism of gas molecule adsorption on two-dimensional ferroelectric materials [33].

In order to understand the mechanism of gas molecule adsorption on the two-dimensional ferroelectric material, single-crystal SnS thin films were used to fabricate the two electrode devices for investigation. Considering that H_2_O is conductive and has high binding energy, resulting in difficulty in desorption, pure NH_3_ with weak binding energy was chosen for the adsorption experiments [34,35,36,37]. Due to the toxicity of CO, NO, and H_2_S, they are not employed for safety considerations. It is found that the NH_3_ molecule’s adsorption and desorption can reversibly regulate the electrical properties of ferroelectric SnS, including I–V curves and transfer curves, etc. After NH_3_ molecules adsorption, the resistance switching state of the I–V hysteresis changed, indicating that NH_3_ adsorption affects the ferroelectric polarizations of SnS. Kelvin probe force microscopy (KPFM) and photo response characterizations indicate that air molecule adsorption significantly impacts the device, and the effect on the contact may be greater than that on the material itself. Additionally, gas sensors fabricated with single-crystal film may have better performance on response time than polycrystalline thin-film gas sensors. These findings are of great significance for the characterization of ferroelectric domains in two-dimensional ferroelectric materials and may promote the gas-sensing applications of two-dimensional ferroelectric materials.

## 2. Materials and Methods

### 2.1. Materials and Characterization

SnS flakes were mechanically exfoliated from the bulk crystal with 3M scotch tape (3M, Minnesota, MN, USA)and then transferred onto a highly p-doped 500 μm Si substrate capped with 285 nm SiO_2_. The SiO_2_/Si (285 nm/500 μm) substrate was used as purchased without any prior treatment. The Cr/Au (5/70 nm) electrodes were deposited onto the flakes to form the source and drain contacts using an electron beam lithography system (Raith Eline Plus, Dortmund, Germany) and an electron beam evaporation system (TECHNOL TEMD500, Beijing, China). The optical images of SnS flakes were obtained from an optical microscope (Nikon ECLIPSE LV 150NL, Tokyo, Japan). The Raman spectra were extracted with a confocal Raman system (Witec Alpha 300R, Ulm, Germany). The AFM, KPFM, and PFM measurements were carried out on a commercial atomic force microscope (NT-MDT, Limerick, Ireland). For the PFM measurements, SnS nanoflakes were transferred onto the 500 μm silica substrate covered with 50 nm fresh Au thin film.

### 2.2. Electrical Measurements

The electrical measurements were carried out in a self-made electrical testing system, as shown in Figure S1, with a vacuum testing box, a gas system, a Keithley 2636B (Cleveland, Ohio, OH, USA), and a LabVIEW control software (https://www.ni.com/zh-cn.html, accessed on 4 May 2024). The response time was measured utilizing a current amplifier and an oscilloscope connected within the circuit. The photocurrent mapping was done in the confocal Raman system (Witec Alpha 300R, Ulm, Germany) with the self-made electrical testing system at 532 nm with a spot size of 700 nm through a 50× objective lens. The photocurrent mapping images were obtained under zero drain voltage and zero gate voltage.

## 3. Results and Discussion

### 3.1. Raman Characterizations of Ferroelectric SnS

**Figure 1:**
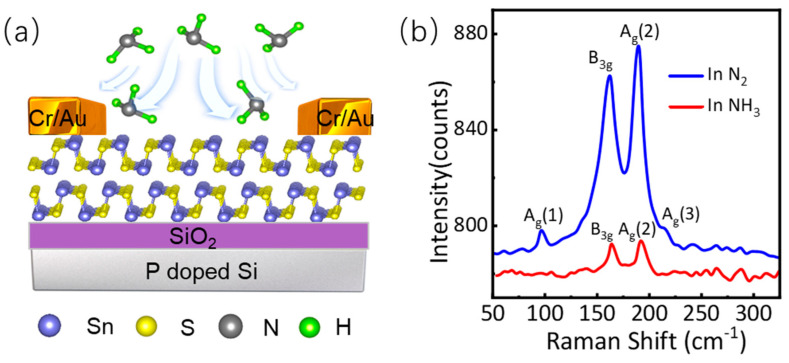
Raman characterization of SnS flake. (**a**) Schematic structure of a ferroelectric SnS device adsorbed with NH_3_ molecules. (**b**) Raman spectra of a few−layered SnS flake in N_2_ and NH_3_.

Figure 1a shows the schematic diagram of a ferroelectric SnS device adsorbed with NH_3_ molecules. Before and after NH_3_ molecules adsorption, the Raman spectra of the thin SnS film were extracted with a confocal Raman system (Witec Alpha 300R, Ulm, Germany). The details about device fabrication and characterization are shown in the method section. Figure 1b shows the Raman spectra of the thin SnS film in N_2_ (blue curve) and NH_3_ (red curve), respectively. The blue curve shows four peaks in its Raman spectrum, located at 95.9 cm^−1^, 163.3 cm^−1^, 191.1 cm^−1^, and 214.2 cm^−1^, which correspond precisely to the four phonon modes A_g_ (1), B_3g_, A_g_ (2), and A_g_ (3) modes of SnS, respectively [17]. The red curve shows that only two peaks are left in its Raman spectrum, located at 163.3 cm^−1^ and 191.1 cm^−1^, which correspond to B_3g_ and A_g_ (2) phonon modes, respectively. The comparison of Raman intensity shows that the Raman peaks of SnS are weakened or disappear by NH_3_ molecules adsorption, indicating that NH_3_ molecules adsorption limits the lattice vibration of SnS. The asymmetric charge distribution, a prominent feature stemming from the lattice structure asymmetry of SnS, is central to its exhibition of ferroelectric properties. Since NH_3_ molecules are polar molecules, their adsorption will reduce the asymmetry of charge distribution in SnS, leading to a decrease in lattice vibration and, consequently, a decrease in Raman Intensity.

### 3.2. Electrical Properties of Ferroelectric SnS

**Figure 2:**
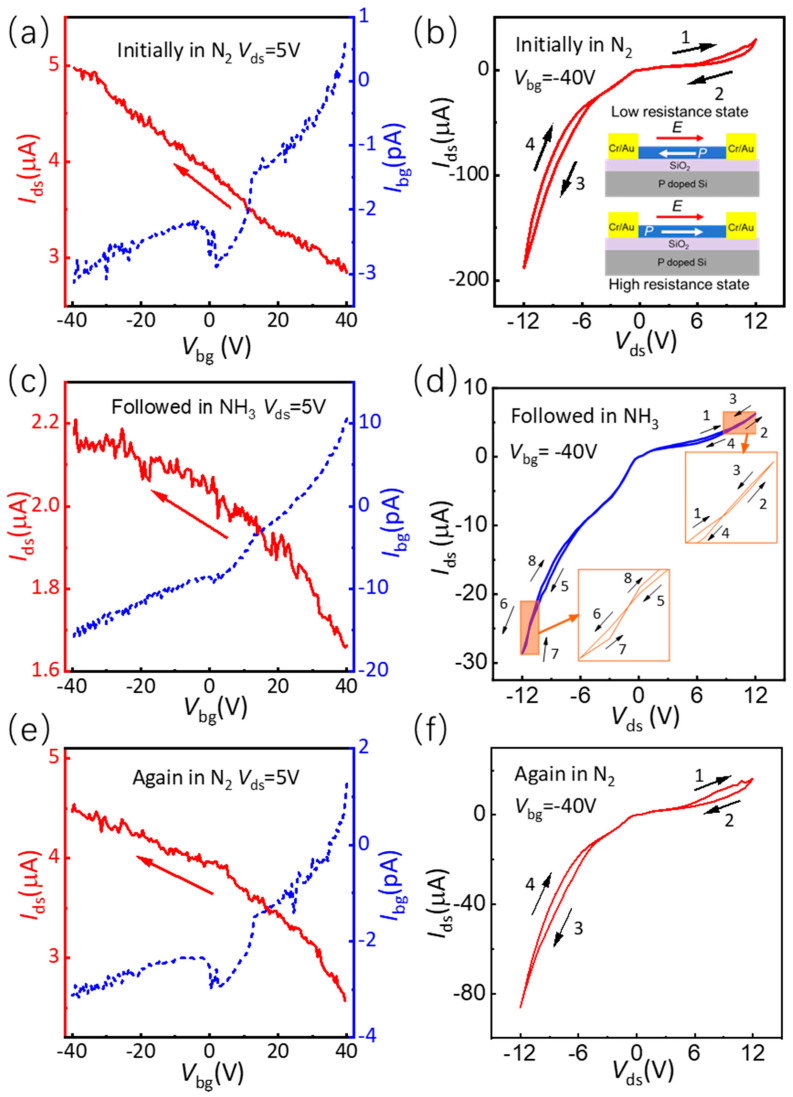
Electrical properties of a few−layered ferroelectric SnS device. (**a**) Initially measured the transfer curve of the device in N_2_. The inset shows the device’s optical image. (**b**) Initially measured I–V hysteresis of the device in N_2_. The inset shows schematic diagrams of the resistance state with different ferroelectric polarizations. (**c**) Followed measured the transfer curve of the device in NH_3_. (**d**) Followed measured the I–V hysteresis of the device in NH_3_. (**e**) Again measured the transfer curve of the device in N_2_. (**f**) Again measured the I–V hysteresis of the device in N_2_. The arrows in the figure indicate the direction of voltage scanning and the dashed line is the back-gate leakage currents of the device.

Electric measurements were carried out in a self-made electrical testing system to analyze the effect of NH_3_ molecule adsorption on the performance of SnS further. Figure 2 shows the electric properties of the ferroelectric SnS device before NH_3_ molecule adsorption, after NH_3_ molecule adsorption, and after NH_3_ molecule desorption. Figure 2a shows the transfer curve of a ferroelectric SnS device in the N_2_ atmosphere. The bias voltage *V*_ds_ was 5 V. When the back gate voltage *V*_bg_ scanned from +40 V to −40 V, the channel current *I*_ds_ increased from 2.85 μA to 4.97 μA, which indicated that the SnS film is hole conductive. The inset of Figure 2a shows the optical image of the ferroelectric SnS device with a channel width and length of about 2.8 μm × 2.1 μm. The thickness of the SnS thin film was determined by atomic force microscope (AFM) characterization to be approximately 82 nm. Based on the size of the device, the hole mobility in the ferroelectric SnS device can be calculated using Equation (1):

(1) $$\mu =\frac{L}{WC_{i}V_{ds}}\frac{dI_{ds}}{dV_{bg}}$$

In the formula, *L* and *W* are the channel length and width of the device, and *C*_i_ is the SiO_2_ dielectric capacitance, which can be calculated from the relative dielectric constant *Ɛ*_r_, the vacuum dielectric constant *Ɛ*_0_, and the dielectric thickness d. *V*_ds_ is the bias voltage. d*I*_ds_/d*V*_bg_ represents the slope extracted from the linear region of the transfer curve. The hole mobility of the ferroelectric SnS device is calculated to be approximately 0.8 cm^2^V^−1^S^−1^. Additionally, the transfer curve of the device appears to be quite noisy, potentially due to low carrier mobility and the testing methodology employed. Given that both the source-drain current and the back-gate current are simultaneously extracted, but the back-gate current is quite small, the extraction process becomes very slow, possibly leading to the high noise observed in the source-drain current. Figure 2b shows the IV hysteresis of the ferroelectric SnS device under −40 V back-gate voltage. Arrows 1, 2, 3, and 4 in the figure show the change direction of the source-drain current in the device. Arrows 1 to 2 and 3 to 4 both indicate that the SnS changes from a low-resistance state to a high-resistance state as the bias voltage increases and then decreases. This is consistent with the previous literature reports [17], and the inset in Figure 2b shows the schematic diagrams of the resistance state with different ferroelectric polarizations. In SnS, the direction of ferroelectric polarization opposes the direction of the electric field as the bias voltage increases and the ferroelectric polarization facilitates carrier transport, leading to a low-resistance state within the device. When the bias voltage increases to a certain level, the direction of ferroelectric polarization is reversed, which is consistent with the direction of the electric field in SnS and hinders carrier transport, leading to a high-resistance state within the device.

Figure 2c shows the transfer curve of the ferroelectric SnS device after NH_3_ molecule adsorption. The bias voltage *V*_ds_ was 5 V. When the back-gate voltage *V*_bg_ scanned from +40 V to −40 V, the channel current *I*_ds_ increased from 1.67 μA to 2.14 μA, confirming that the SnS film is still hole conductive. The hole mobility of SnS was calculated to be approximately 0.2 cm^2^V^−1^S^−1^ using the former calculation method. The results showed that the concentration and mobility of SnS holes decreased after NH_3_ molecule adsorption, indicating that the SnS is electron-doped with NH_3_ molecule adsorption. Figure 2d shows the IV hysteresis of the ferroelectric SnS device after adsorbing NH_3_ molecules under −40 V back-gate voltage. Arrows 1 to 8 in the figure show the direction of channel current variation with bias voltage in SnS. Crossings are observed in arrows 1 to 4 and 5 to 8, and the local magnifications of the crossing positions are shown in the inset of Figure 2d. Compared with the resistance switching state in Figure 2b, the observed crossings and smaller ratio in a high-resistance and a low-resistance indicated that the ferroelectric polarization in SnS is weakened with NH_3_ molecule adsorption and maybe un-uniform in the device. To further understand the adsorption mechanism, the NH_3_ molecule adsorption is substituted with the N_2_ molecule adsorption by continuously injecting N_2_ into the electrical testing box. Figure 2e,f show the transfer curve and IV hysteresis of the ferroelectric SnS device, respectively, remeasured in an N_2_ atmosphere. The bias voltage *V*_ds_ is 5 V. As shown in Figure 2e, when the back-gate voltage *V*_bg_ scanned from +40 V to −40 V, the channel current *I*_ds_ increased from 2.57 μA to 4.46 μA. The hole mobility of SnS can be calculated to be approximately 0.7 cm^2^V^−1^S^−1^ using the former calculation method. Figure 2f shows the IV hysteresis of the ferroelectric SnS device after NH_3_ molecule desorption under −40 V back gate voltage. Arrows 1 to 4 in the figure show the channel current variation direction with bias voltage in SnS. In comparison to the results presented in Figure 2a,b, the reintroduction of the N_2_ reveals that the hole concentration and mobility of SnS have been nearly fully recovered, with the resistance switching state also returning to its original condition. This observation suggests that NH_3_ molecule adsorption and desorption could reversibly tune the electric properties of the ferroelectric SnS device.

**Figure 3:**
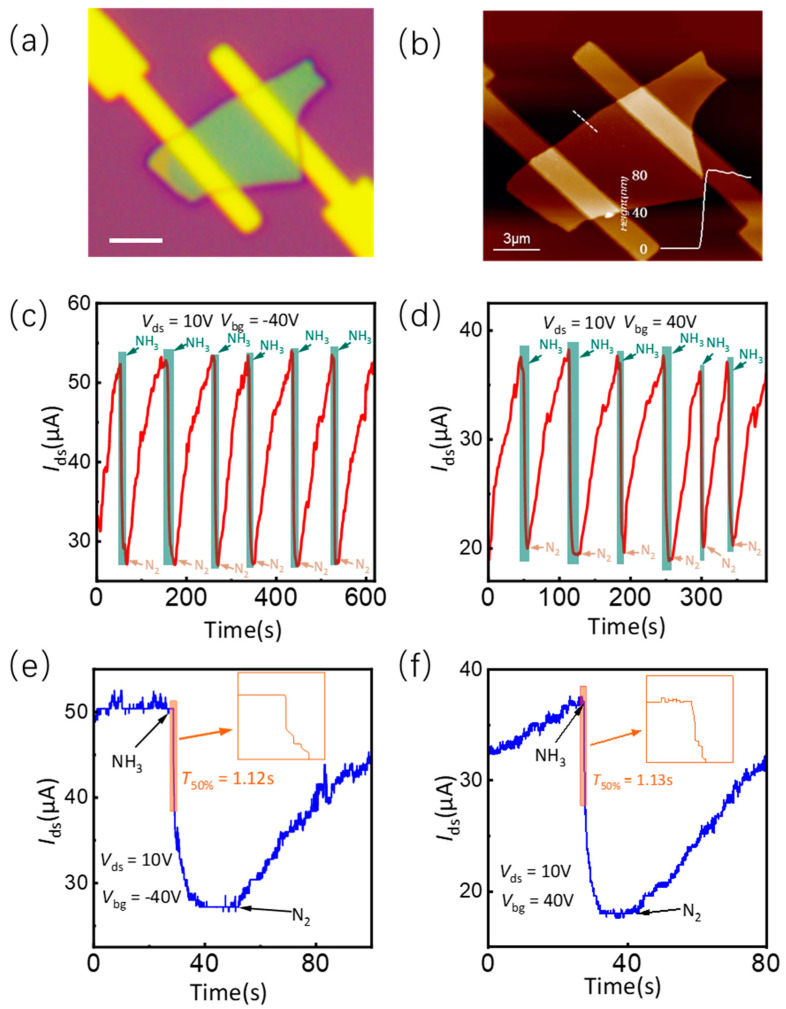
Time response of the ferroelectric SnS device. (**a**) Optical image of the ferroelectric SnS device. The scale bar is 4 μm. (**b**) AFM image of the ferroelectric SnS device. The inset shows the height profile of the white dashed line. Dynamic response of the ferroelectric SnS device at *V*_bg_ = −40 V (**c**) and *V*_bg_ = 40 V (**d**). Response time of the ferroelectric SnS device at *V*_bg_ = −40 V (**e**) and *V*_bg_ = 40 V (**f**). The inset shows a magnified view of the current reaching 50% of the steady state.

To further confirm the reversible modulation of the electrical properties of SnS by NH_3_ molecule adsorption and desorption, the cyclic on-off response was measured by sequentially alternating between NH_3_ adsorption and N_2_ purging. Figure 3a shows the optical image of the device with metal electrodes of Cr/Au (5/70 nm). Figure 3b shows the topography image of the device with AFM characterization. The inset in Figure 3b displays the height profile measured along the white dash line in the figure, revealing a film thickness of approximately 80 nm. When NH_3_ and N_2_ are sequentially introduced at a flow rate of 0.5 standard liters per minute (SLM), Figure 3c,d show the channel current *I*_ds_ response of the ferroelectric SnS device under −40 V and +40 V back-gate voltage, respectively. The green-shaded region within the figure corresponds to the operational state of the device during the passage of NH_3_, whereas the remaining area represents the device state during the subsequent passage of N_2_. It is obvious that the channel current of the device undergoes a decrease when NH_3_ is introduced and subsequently recovers to its original level upon the introduction of N_2_. This confirmed that the resistance of the device is reversibly tuned with NH_3_ adsorption and desorption. The sensitivity of the SnS thin film to NH_3_ molecules can be analyzed by calculating the responsivity *R*, according to Equation (2).

(2) $$R=\frac{R_{G}}{R_{A}}$$

In the formula, *R*_G_ and *R*_A_ represent the resistance values of the ferroelectric SnS device when exposed to NH_3_ and N_2_ atmospheres, respectively. As shown in Figure 3c, under a back-gate voltage of −40 V and a bias voltage of +10 V, as NH_3_ and N_2_ are sequentially introduced at a flow rate of 0.5 SLM, the responsivities of the device over six consecutive cycles are found to be 1.97, 2.00, 1.99, 2.03, 1.98, and 2.01, respectively. As shown in Figure 3d, under a back-gate voltage of +40 V and a bias voltage of +10 V, as NH_3_ and N_2_ are sequentially introduced at a flow rate of 0.5 SLM, the responsivities of the device over six consecutive cycles are found to be 1.98, 1.99, 2.02, 1.83, 1.95, and 1.98, respectively. The analysis reveals a maximum deviation in responsivity of approximately 7.3%, suggesting that the utilization of NH_3_ molecule adsorption and desorption as a means to modulate the electrical properties of SnS exhibits commendable reversibility and repeatability. Since the amount of ammonia gas influx is manually adjusted in real-time, it is predictable that the adsorption and desorption of ammonia gas molecules can cause the SnS resistance to vary within a certain range. Figure 3e,f show a single on-off response of the ferroelectric SnS device to NH_3_ with a 0.5 SLM rate at −40 V and 40 V back-gate voltage, respectively. The single on-off response is extracted via the oscilloscope observation connected to the circuit. The inset of Figure 3e presents a magnified view of the response, demonstrating that the device generates a response within 20 ms, with a time duration of 1.12 s to attain a stable response level of 50%. The recovery process requires a relatively prolonged duration of approximately 55 seconds. Due to the employment of alternating NH_3_ adsorption coupled with N_2_ ventilation, it is logical to expect a relatively extended duration for the complete desorption of NH_3_ molecules. The utilization of a vacuum system might accelerate the recovery process. The inset featured in Figure 3f exhibits an analogous phenomenon to that presented in Figure 3e. As shown in the Supplementary Table S1, in contrast to gas sensors based on other two-dimensional materials [38,39,40,41,42], such as black phosphorus, utilizing ferroelectric thin film exhibits significantly faster response speed.

### 3.3. The Mechanism of NH_3_ Molecule Adsorption

**Figure 4:**
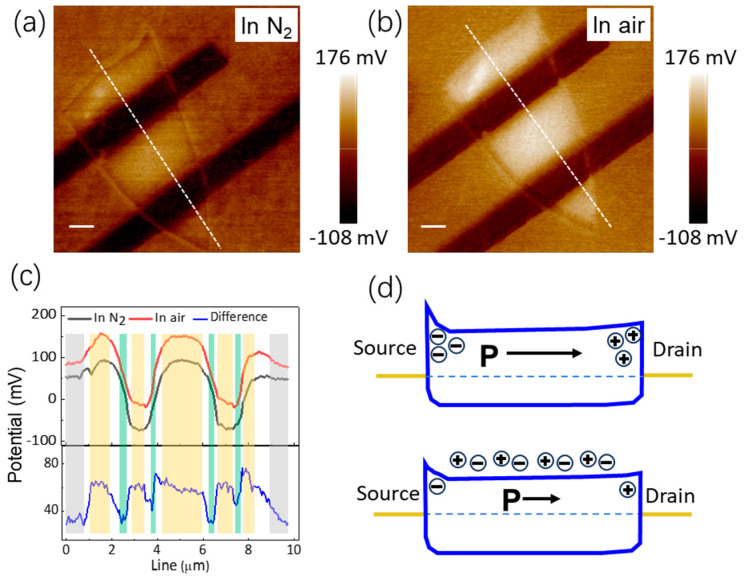
KPFM images of a ferroelectric SnS device. (**a**) In N_2_. (**b**) In air. The scale bar is 1 μm. The white dashed lines indicate where the surface potential is extracted. (**c**) Extracted potentials and their difference at the white dashed line in (**a**,**b**). (**d**) Illustration of the band diagrams of the device with and without air molecule adsorption.

In order to elucidate the mechanism of NH_3_ molecule adsorption on the device, Kelvin probe force microscopy (KPFM) measurements were performed. Due to the lack of an atmospheric environment in the AFM system, it is difficult to obtain KPFM images in an NH_3_ atmosphere. As shown in Figure S3, air molecules have similar effects as NH_3_ on the electrical properties of the ferroelectric SnS device, and KPFM images were extracted in N_2_ and air atmospheres, respectively, to analyze the mechanism of molecule adsorption. The KPFM images prior to and subsequent to air molecule adsorption are depicted in Figure 4a,b, respectively. Upon comparing Figure 4a,b, it is evident that the surface potentials of SnS material, metal electrode, and SiO_2_ sheet all undergo subsequent modifications to the introduction of air, with varying degrees of magnitude in these changes. For quantitative analysis, the surface potential values from identical locations were precisely extracted within N_2_ and air atmospheres. The specific extraction positions are clearly indicated by the white dashed lines depicted in Figure 4a,b. These positions traverse through the layers in the following sequence: SiO_2_, SnS, Cr/Au, SnS, Cr/Au, SnS, and finally ending with SiO_2_. This methodology ensures an accurate comparison of surface potential variations across different environments. The surface potential results are shown in Figure 4c. A comparative evaluation reveals that the surface potentials of SiO_2_, SnS, and Cr/Au electrodes underwent notable increases of 37 mV, 63 mV, and 61 mV, respectively. This indicates a similar yet slightly stronger influence of air on SnS and Cr/Au, in contrast to a marginally weaker effect on SiO_2_. Thus, the air’s effect is more pronounced for SnS and Cr/Au materials. Furthermore, an intriguing observation was made regarding the surface potential variation at the interface between SnS and Cr/Au. Notably, fluctuations in the height at this contact point suggest potential errors or anomalies occurring precisely at this junction. Therefore, the band structure of SnS devices can be examined, as illustrated in Figure 4d. In an N_2_ environment, the ferroelectric polarization accumulates varied charges at the source and drain electrode interfaces, leading to the formation of an asymmetric electrode contact configuration. Given the hole-conductive nature of SnS, when the direction of the ferroelectric polarization (P) opposes that of the biased electric field, the accumulation of polarized charges from the ferroelectric material weakens the built-in electric field at the drain contact while strengthening it at the source contact. Upon the adsorption of air molecules, the ferroelectric polarization intensity diminishes, resulting in a weakening of the contact electric field at both ends. Consequently, the elevated surface potential further lowers the hole concentration, ultimately diminishing the conductivity of the device.

**Figure 5:**
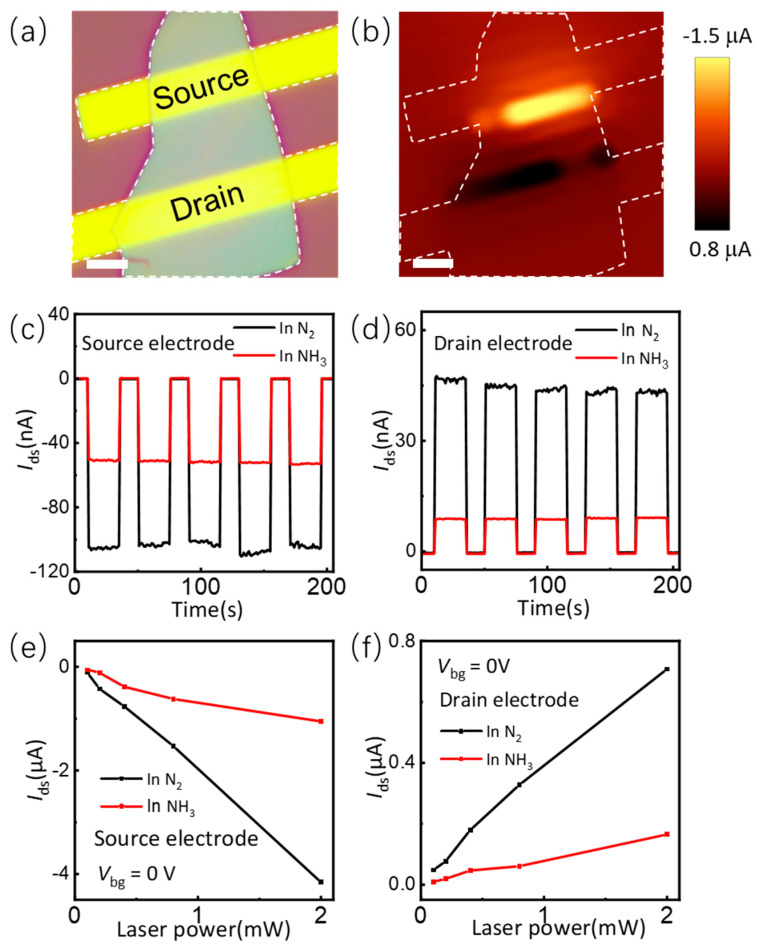
Photo response of the ferroelectric SnS device. (**a**) Optical image of the ferroelectric SnS device. The scale bar is 3 μm. (**b**) Photocurrent image of the ferroelectric SnS device at zero bias voltage. (**c**) On−off photo response of the device at source electrode in N_2_ and NH_3_. (**d**) On−off photo response of the device at drain electrode in N_2_ and NH_3_. (**e**) Photo response at source electrode in N_2_ and NH_3_ verse laser power. (**f**) Photo response at drain electrode in N_2_ and NH_3_ verse laser power.

As the SnS film possesses an indirect bandgap of 1.1 eV [17,23], it exhibits a remarkable light absorption coefficient for both visible and near-infrared light. Hence, the adsorption of NH_3_ molecules is likely to influence its photoelectric response characteristics. To analyze the changes in its photoelectric performance subsequent to NH_3_ molecule adsorption, photo response characterizations of the device were carried out in the Witec Alpha 300R Raman system. As shown in Figure 5a, this is an optical photograph of the ferroelectric SnS device with a scale bar of 3 μm. Figure 5b corresponds to a scanning photocurrent image of the device. The photocurrent image reveals that the photoelectric response of SnS devices, under zero bias conditions, is predominantly localized at the electrode interface where Cr/Au contacts SnS. This suggests the formation of a Schottky contact between Cr/Au and SnS, influencing the device’s photoelectric behavior. The observation of positive photocurrent at the Drain contact and negative photocurrent at the Source contact uncovers the hole-conducting nature of SnS. Notably, the higher photocurrent at the Source contact compared to the Drain contact hints at the presence of asymmetric contacts. This asymmetry aligns seamlessly with the accumulation of ferroelectric polarization charges, with the ferroelectric polarization direction being in opposition to the direction of the applied bias electric field. Figure 5c,d present the photoelectric responses of the Source and Drain contacts, respectively, under N_2_ and NH_3_ atmospheric conditions. The obtained results align well with the photocurrent image, demonstrating a notable reduction in the photo responsivity by NH_3_ molecule adsorption. This reduction is in harmony with the diminished ferroelectric polarization intensity, the weakened built-in electric field at the contact interface, and the decreased hole concentration within the device, all of which occur upon the adsorption of NH_3_ molecules. Figure 5e,f present the photoelectric responses of the device at the Source and Drain contacts, respectively, under varying laser powers in both N_2_ and NH_3_ atmospheres. The findings indicate that as the laser power increases, the photoelectric response becomes increasingly linear. Notably, following the adsorption of NH_3_ molecules, the photoelectric response at the Source contact diminishes substantially, from approximately −2 mA/W to −0.5 mA/W, while the response at the Drain contact also undergoes a reduction from around 0.4 mA/W to 0.1 mA/W.

## 4. Conclusions

This study delves into the impact of NH_3_ molecule adsorption on the ferroelectric SnS, revealing its ability to reversibly modulate its electrical properties. Specifically, NH_3_ adsorption switches the IV hysteresis direction from low to high resistance states and even a cross-state, with full reversibility upon desorption. Notably, this transition occurs swiftly, with a 50% response time of approximately 1.13 s and a response rate better than 20 ms. KPFM characterizations indicate that NH_3_ adsorption alters the surface potentials of SiO_2_, SnS, metal electrodes, and contacts with minimal impact on the contact surface potential. Upon the adsorption of NH_3_ molecules, the intensity of ferroelectric polarization diminishes, and the hole concentration within the device decreases. Consequently, the photoelectric responses at the electrode contacts are reduced. These findings are of great significance for the characterization of ferroelectric domains in two-dimensional ferroelectric materials and may promote the gas-sensing applications of two-dimensional ferroelectric materials.

## Data Availability

The original contributions presented in the study are included in the article/Supplementary Material, further inquiries can be directed to the corresponding author.

## Supplementary Materials

The following supporting information can be downloaded at: https://www.mdpi.com/article/10.3390/nano14201638/s1. Figure S1: The schematic diagram of the self-made electrical measure system, Figure S2: Figures of PFM measurements of SnS flake, Figure S3: electric properties of a few-layered ferroelectric SnS device, Table S1: the response time of gas sensors based on other two−dimensional materials [35,43,44,45], Figure S4: photo response of a few-layered ferroelectric SnS device, and Figure S5: I–V curves of a few-layered ferroelectric SnS device.
