# Supplementary material for: Reversible Tuning Electrical Properties in Ferroelectric SnS with NH3 Adsorption and Desorption
Source: Nanomaterials (Basel). 2024 Oct 12;14(20):1638. doi: 10.3390/nano14201638 (PMC11510606; doi:10.3390/nano14201638)
Supplement: Supplementary file 1 [file nanomaterials-14-01638-s001.zip › nanomaterials-3238307-supplementary.pdf]

Supporting Information

Reversible tuning electrical properties in ferroelectric SnS with NH_3 adsorption and desorption

Wanqian Wang, [#] *Wei Luo,* [#,*] *Sen Zhang, Chayuan Zeng, Fei Xie, Chuyun Deng, and Gang Peng,*[*]

College of Science, National University of Defense Technology, Changsha 410073, China

*Corresponding authors

Email: luowei@nudt.edu.cn

Email: penggang@nudt.edu.cn

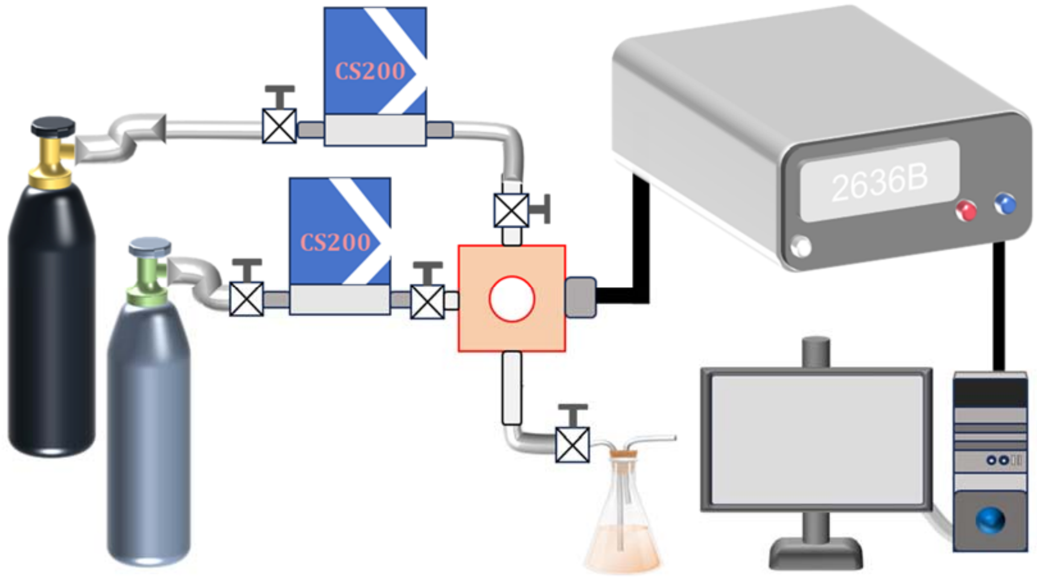

Figure S1 The schematic diagram of the self-made electrical measure system

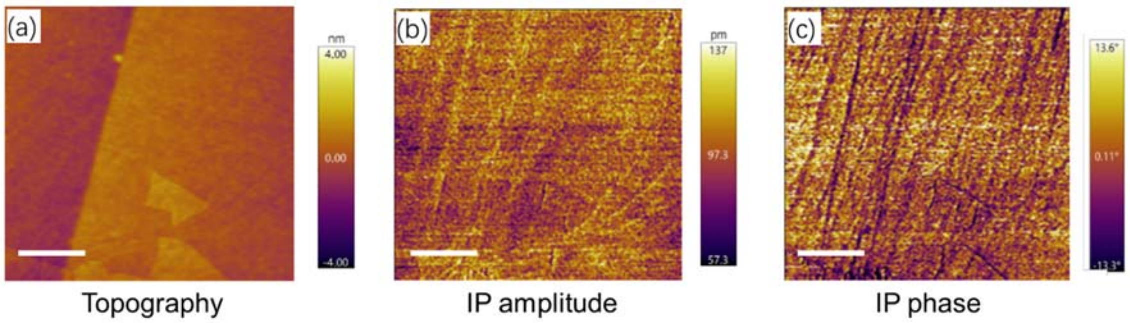

Figure S2. PFM measurements of SnS flake. (a) Topography. (b) In-plane (IP) amplitude. (c) IP phase. The scale bar is 1 μm.

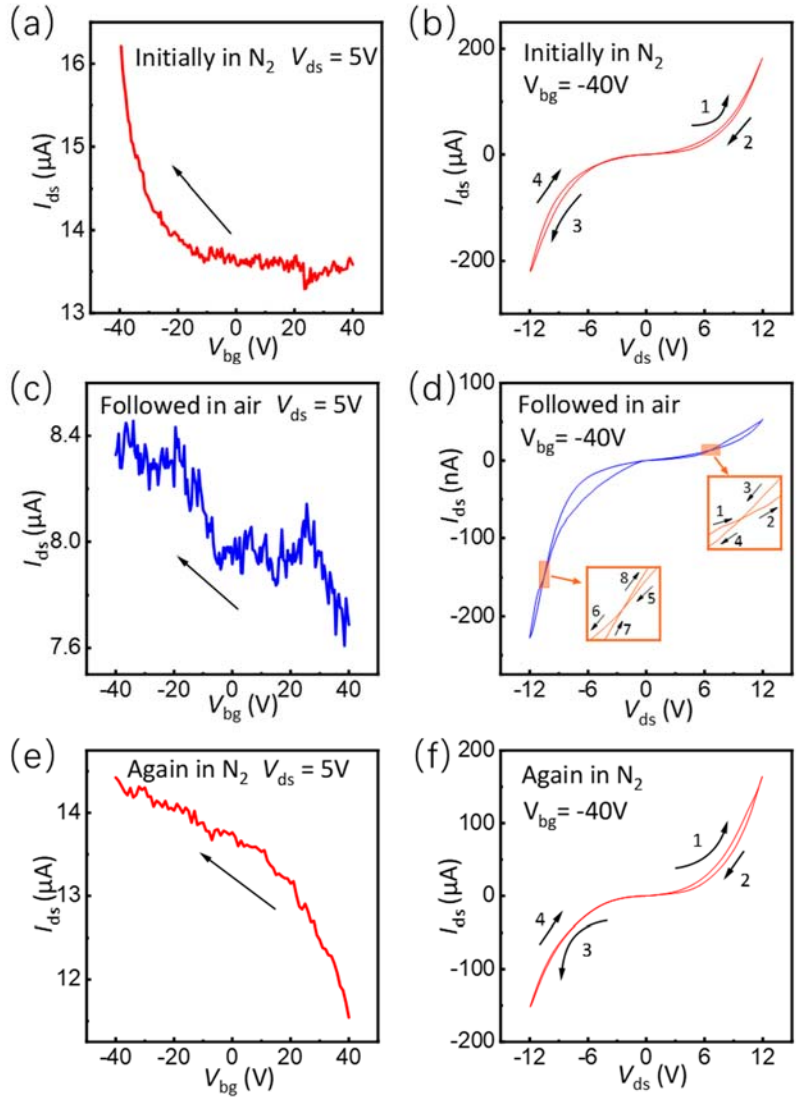

Figure S3. Electric properties of a few-layer SnS device. (a) Initially measured transfer curve of the device in N_2. (b) Initially measured IV hysteresis of the device in N_2. (c) Followly measured transfer curve of the SnS device in NH_3. (d) Followly measured IV hysteresis of the SnS device in NH_3. (e) Againly measured transfer curve of the SnS device in N_2. (f) Againly measured IV hysteresis of the SnS device in N_2.

Sensing Materials	Target Gas	Sensing Concentration	Response Time	Ref.
1T/2H-MoS_2/graphene	NH_3	100~400 ppm	186~407 s	[1]
multilayer BP	NO_2	5~40 ppb	130~840 s	[2]
$PtSe_2$	NH_3	2000 ppm	115 s/234 s	[3]
MoS_2 FET	NH_3	1~500 ppm	5~9 min	[4]

Table 1 The response time of gas sensors based on other two-dimensional materials

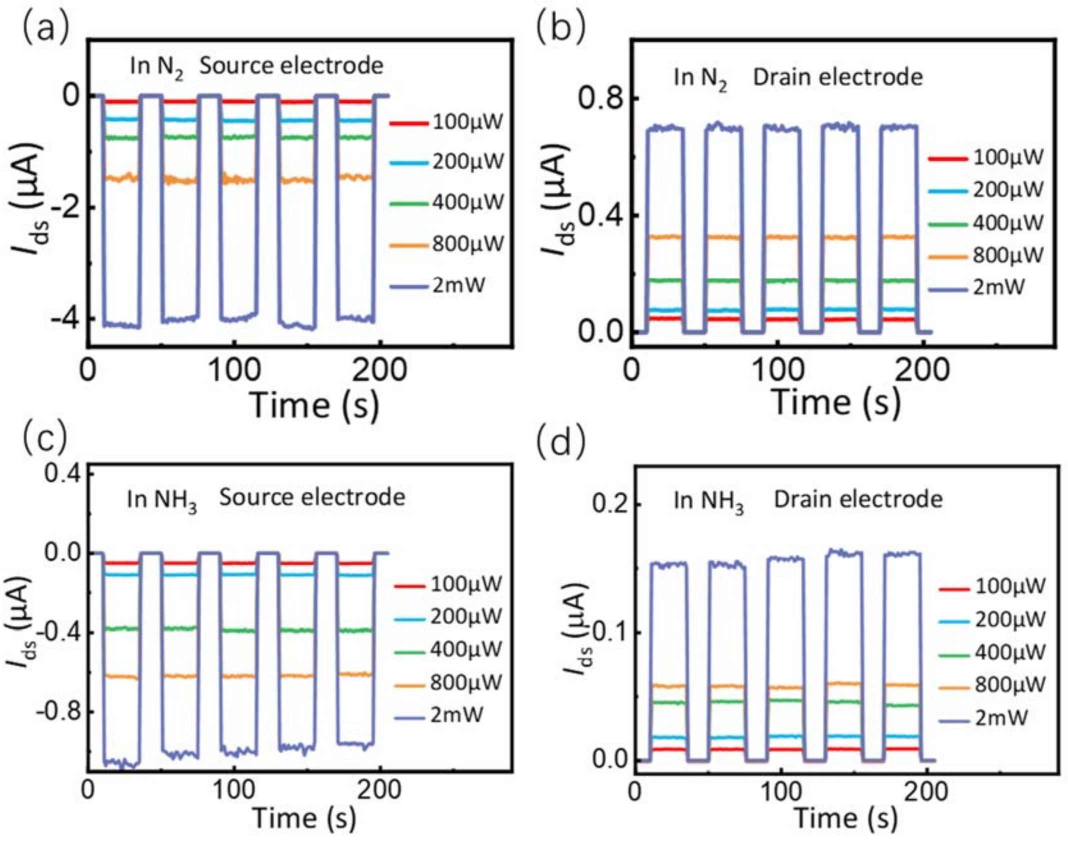

Figure S4. Photo response of a few-layer SnS device. (a) On-off response at source electrode in N_2 with different laser power. (b) On-off response at drain electrode in N_2 with different laser power. (c) On-off response at source electrode in NH_3 with different laser power. (d) On-off response at drain electrode in NH_3 with different laser power. V_{bg} =0V. V_{ds} = 0V. The laser wavelength is 532 nm.

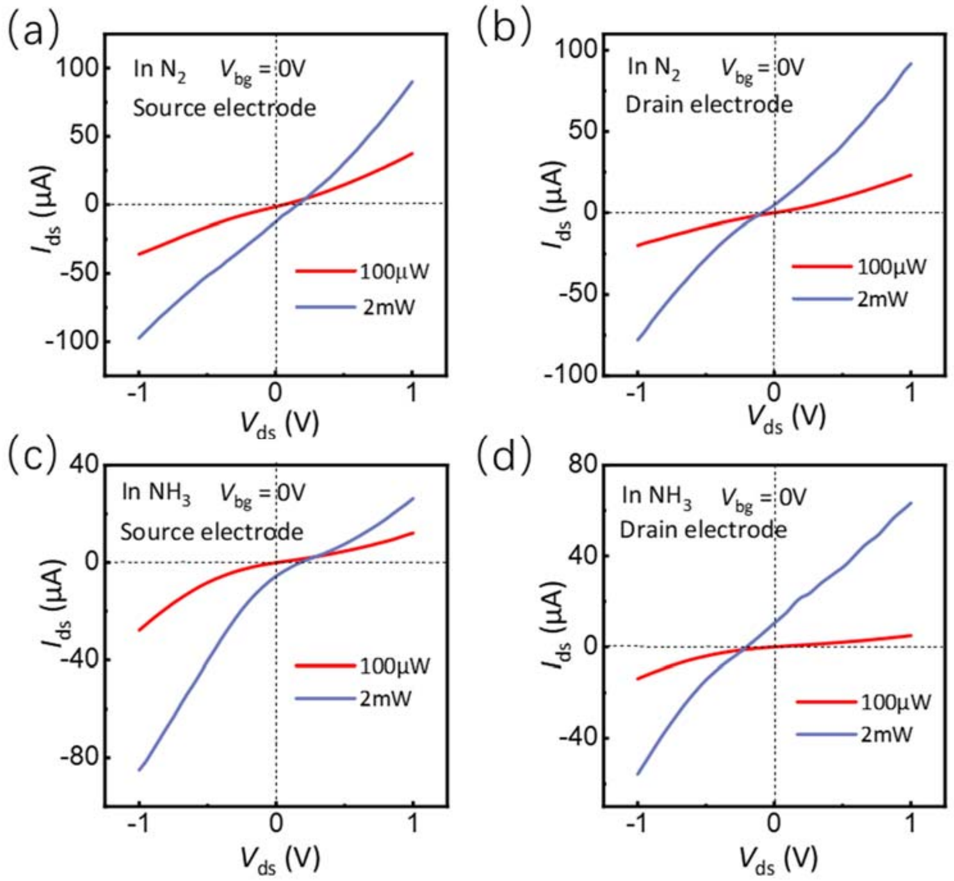

Figure S5. IV curves of a few-layer SnS device. (a) IV curves in N_2 with different focused laser power illuminating at source electrode. (b) IV curves in N_2 with different focused laser power illuminating at drain electrode. (c) IV curves in NH_3 with different focused laser power illuminating at source electrode. (d) IV curves in NH_3 with different focused laser power illuminating at drain electrode.

Reference

[1] Zhang, C.; Ning, J.; Wang, B.; Guo, H.; Feng, X.; Shen, X.; Jia, Y.; Dong, J.; Wang, D.; Zhang, J.; et al. Hybridized 1T/2H-MoS2/graphene fishnet tube for high-performance on-chip integrated micro-systems comprising supercapacitors and gas sensors. Nano Research 2021, 14 (1), 114-121. DOI: 10.1007/s12274-020-3052-x.

[2] Abbas, A. N.; Liu, B.; Chen, L.; Ma, Y.; Cong, S.; Aroonyadet, N.; Köpf, M.; Nilges, T.; Zhou, C. Black Phosphorus Gas Sensors. ACS Nano 2015, 9 (5), 5618-5624. DOI: 10.1021/acsnano.5b01961.

[3] Wang, Z.; Jing, X.; Duan, S.; Liu, C.; Kang, D.; Xu, X.; Chen, J.; Xia, Y.; Chang, B.; Zhao, C.; et al. 2D PtSe2 Enabled Wireless Wearable Gas Monitoring Circuits with Distinctive Strain-Enhanced Performance. ACS Nano 2023, 17 (12), 11557-11566. DOI: 10.1021/acsnano.3c01582.

[4] Liu, B.; Chen, L.; Liu, G.; Abbas, A. N.; Fathi, M.; Zhou, C. High-Performance Chemical Sensing Using Schottky-Contacted Chemical Vapor Deposition Grown Monolayer MoS2 Transistors. ACS Nano 2014, 8 (5), 5304-5314. DOI: 10.1021/nn5015215.
